# Discovery of Markers of Exposure Specific to Bites of *Lutzomyia longipalpis*, the Vector of *Leishmania infantum chagasi* in Latin America

**DOI:** 10.1371/journal.pntd.0000638

**Published:** 2010-03-23

**Authors:** Clarissa Teixeira, Regis Gomes, Nicolas Collin, David Reynoso, Ryan Jochim, Fabiano Oliveira, Amy Seitz, Dia-Eldin Elnaiem, Arlene Caldas, Ana Paula de Souza, Cláudia I. Brodskyn, Camila Indiani de Oliveira, Ivete Mendonca, Carlos H. N. Costa, Petr Volf, Aldina Barral, Shaden Kamhawi, Jesus G. Valenzuela

**Affiliations:** 1 Vector Molecular Biology Section, Laboratory of Malaria and Vector Research, National Institute of Allergy and Infectious Diseases, National Institutes of Health, Rockville, Maryland, United States of America; 2 Department of Natural Sciences, University of Maryland Eastern Shore, Princess Anne, Maryland, United States of America; 3 Universidade Federal do Maranhão, São Luis, Maranhão, Brazil; 4 Centro de Pesquisas Goncalo Moniz–FIOCRUZ, Salvador, Bahia, Brazil; 5 Laboratorio de Leishmanioses, Instituto de Doencas Tropicais Natan Portella and Universidade Federal do Piaui, Teresina, Piaui, Brazil; 6 Department of Parasitology, Faculty of Science, Charles University in Prague, Prague, Czech Republic; Institut Pasteur, France

## Abstract

**Background:**

Sand flies deliver *Leishmania* parasites to a host alongside salivary molecules that affect infection outcomes. Though some proteins are immunogenic and have potential as markers of vector exposure, their identity and vector specificity remain elusive.

**Methodology/Principal Findings:**

We screened human, dog, and fox sera from endemic areas of visceral leishmaniasis to identify potential markers of specific exposure to saliva of *Lutzomyia longipalpis.* Human and dog sera were further tested against additional sand fly species. Recombinant proteins of nine transcripts encoding secreted salivary molecules of *Lu. longipalpis* were produced, purified, and tested for antigenicity and specificity. Use of recombinant proteins corresponding to immunogenic molecules in *Lu. longipalpis* saliva identified LJM17 and LJM11 as potential markers of exposure. LJM17 was recognized by human, dog, and fox sera; LJM11 by humans and dogs. Notably, LJM17 and LJM11 were specifically recognized by humans exposed to *Lu. longipalpis* but not by individuals exposed to *Lu. intermedia*.

**Conclusions/Significance:**

Salivary recombinant proteins are of value as markers of vector exposure. In humans, LJM17 and LJM11 emerged as potential markers of specific exposure to *Lu. longipalpis*, the vector of *Leishmania infantum chagasi* in Latin America. In dogs, LJM17, LJM11, LJL13, LJL23, and LJL143 emerged as potential markers of sand fly exposure. Testing these recombinant proteins in large scale studies will validate their usefulness as specific markers of *Lu. longipalpis* exposure in humans and of sand fly exposure in dogs.

## Author Summary

*Leishmania* parasites are transmitted by the bite of an infected vector sand fly that injects salivary molecules into the host skin during feeding. Certain salivary molecules can produce antibodies and can be used as an indicator of exposure to a vector sand fly and potentially the disease it transmits. Here we identified potential markers of specific exposure to the sand fly *Lutzomyia longipalpis*, the vector of visceral leishmaniasis in Latin America. Initially, we determined which of the salivary proteins produce antibodies in humans, dogs, and foxes from areas endemic for the disease. To identify potential specific markers of vector exposure, we produced nine different recombinant salivary proteins from *Lu. longipalpis* and tested for their recognition by individuals exposed to another human-biting sand fly, *Lu. intermedia*, that transmits cutaneous leishmaniasis and commonly occurs in the same endemic areas as *Lu. longipalpis*. Two of the nine salivary proteins were recognized only by humans exposed to *Lu. longipalpis*, suggesting they are immunogenic proteins and may be useful in epidemiological studies. The identification of specific salivary proteins as potential markers of exposure to vector sand flies will increase our understanding of vector–human interaction, bring new insights to vector control, and in some instances act as an indicator for risk of acquiring disease.

## Introduction

Sand fly salivary proteins play a major role in blood feeding and Leishmania transmission [1]–[3]. Exposure to sand fly salivary proteins induces both cellular immunity and specific antibodies [3],[4]. A relationship between the level of specific antibodies to saliva, vector exposure and risk of contracting disease has been demonstrated for different vector-host models [5]–[9]. Production of antibodies against mosquito and tick saliva not only contributed to development of host allergic reactions but was strongly related to risk of disease development [5],[10]. Similarly, in an endemic area of Senegal, production of antibodies against *Anopheles gambiae* salivary proteins was identified as an indicator of the risk of malaria [10]. This correlation was also observed for tick exposure, where antibody production against tick saliva was associated with self-reported tick exposure and Lyme disease [11]. Recently, saliva of *Triatoma infestans* was shown to be a potential marker for vector infestation in domestic animals [12]. Therefore, the detection of antibodies against the saliva of hematophagous insect vectors could be used as an indicator of vector exposure and in some instances as an indicator for risk of contracting disease.

Previous work shows that humans and animals exposed to sand fly bites or immunized with saliva can develop antibodies that recognize specific salivary proteins [4], [7], [13]–[15]. In São Luis, an area of endemic visceral leishmaniasis (VL) in Maranhão, Brazil, the presence of anti-saliva antibodies in humans strongly correlated with protection and the development of anti-*Leishmania* delayed-type hypersensitivity response [7]. Furthermore, individuals that poorly recognized salivary proteins developed anti-*Leishmania* antibodies associated with disease progression [7]. In contrast, in areas endemic for cutaneous leishmaniasis (CL)—such as Canoa (Bahia, Brazil) and Sanliurfa (Turkey)—the presence of anti-saliva antibodies correlated with risk of contracting disease [16],[17].

The presence of antibodies to sand fly salivary proteins has also been demonstrated in animal reservoirs of leishmaniasis. In canines, two sand fly salivary proteins were recognized by sera of infected dogs from an endemic VL area in Brazil [18]. Hostomska et al. [14] reported the presence of anti-saliva antibodies to six different sand fly proteins in dogs experimentally exposed to *Lutzomyia longipalpis* bites. Importantly, foxes captured in Teresina, an endemic VL area in Brazil, also showed high levels of anti-saliva antibodies, particularly to a 44-kDa salivary protein from *Lu. longipalpis*, suggesting exposure to bites of this vector [19]. Hence, vector salivary proteins also represent a potential tool as markers of exposure to important reservoirs of disease.

Identification of the sand fly salivary proteins recognized by the mammalian host will not only increase our understanding of vector-host interactions but will also aid in developing new epidemiological tools to correlate host exposure to vector sand flies with immunity or susceptibility to leishmaniasis. It will also help identify potential reservoirs of Leishmania. Here we describe a practical functional transcriptomic approach for the identification of the *Lu. longipalpis* salivary proteins most recognized by humans and canids (dogs and foxes) using sera from São Luis and Teresina, endemic areas for VL in Brazil [15],[20].

## Methods

### Sand flies and preparation of salivary gland homogenate (SGH)

*Lu. longipalpis* (Jacobina strain) were reared at LMVR, NIAID, USA; *Lu. verrucarum* (Peru strain) and *Phlebotomus perniciosus* (Italy strain) at WRAIR, USA; *Lu. intermedia* (Corte de Pedra strain) were obtained from CPqGM (FIOCRUZ, Bahia, Brazil). Females were used for dissection of salivary glands 5–8 days post-eclosion; SGH was prepared as described elsewhere [21]. Briefly, salivary glands were dissected and stored in sterile PBS (pH 7.4) at −70°C. To obtain the homogenate, salivary glands were disrupted by ultrasonication and the supernatant collected after centrifugation at 15,000g for 2 minutes.

### Serum samples

A total of 14 human sera from from a VL-endemic region in São Luis (Maranhão, Brazil) [15] and 6 from a CL-endemic region in Canoa (Bahia, Brazil) [22] were used in this study. Informed written consent was obtained from parents or legal guardians of minors. The project was approved by the institutional review board from the Federal University of Bahia (1993) and the Federal University of Maranhao (1996). Dog and fox (*Cerdocyon thous*) sera (total of 8 and 11, respectively) were from animals captured in a VL-endemic area around Teresina (Piaui, Brazil) [19]. Fox and dog studies were approved in 2000 by the Brazilian agency for protection of the wildlife (IBAMA/PI) and in 2005 by the Federal University of Piaui. Sera were also obtained from dogs (total of 6) experimentally exposed to *Lu. longipalpis* [14]. Dog studies were approved by Bayer Health Care AG (Leverkusen, Germany) and handled in accordance with the European guidelines for animal husbandry.

### Cloning of *Lu. longipalpis* salivary transcripts into a VR2001-TOPO vector

DNA was amplified by polymerase chain reaction (PCR) using a forward primer deduced from the amino-terminus and a reverse primer encoding a hexhistidine motif. PCR amplification conditions were: one hold of 94°C 5 min, two cycles of 94°C 30 s, 48°C 1 min, 72°C 1 min, 23 cycles of 94°C 30 s, 58°C 1 min, 72°C 1 min, and one hold of 72°C 7 min. The PCR product was cloned into the VR2001-TOPO vector and sequenced [23].

### Polyclonal antibodies against *Lu. longipalpis* salivary protein

A plasmid encoding a distinct salivary protein (1 µg/µl) was injected intradermally into female Swiss Webster mice in 10 µl, three times at two-week intervals to generate polyclonal antibodies for each of the nine selected candidates [23].

### Expression and high-performance liquid chromatography (HPLC) purification of His-tagged *Lu. longipalpis* salivary proteins

Recombinant proteins were produced by transfecting 293-F cells (Invitrogen) with plasmid following the manufacturer's recommendations. After 72 h, the supernatant was recovered, filtered and concentrated to 30 ml in an Amicon concentrator device (Millipore) in the presence of Buffer A (20 mM NaH_2_PO_4_, 20 mM Na_2_HPO_4_, pH 7.4, 500 mM NaCl). A HiTrap chelating HP column (GE Healthcare) was charged with 5 ml of 0.1M Ni_2_SO_4_. The concentrated protein was added to the HiTrap chelating HP column that was then connected to a Summit station HPLC system (Dionex, Sunnyvale, CA) consisting of a P680 HPLC pump and a PDA-100 detector. The column was equilibrated for 30 min with Buffer A at 1 ml/min. Elution conditions were: 0-5 min, 100% Buffer A; 5-15 min, a gradient of 0% to 100% Buffer B ( Buffer A+50 mM imidazole); 15-20 min, a gradient of 0% C (Buffer A+500 mM imidazole) to 10% C (90% B); 20-25 min, 90% B and 10% C; min 25-30, a gradient of 10% C to 20% C (80% B); 30-35 min, 80% B and 20% C; 35-40 min, a gradient of 20% C to 100%C; and 40-50 min, 100% C. Eluted proteins were detected at 280 nm and collected every minute on a 96-well microtiter plate using a Foxy 200 fraction collector (Teledyne ISCO).

Five-microliter aliquots of all fractions were blotted on nitrocellulose and blocked with TBS-tween 3% non-fat milk for 1 h and then incubated for 1 h with anti-saliva antibodies, washed, and incubated for 1 h with an anti-mouse IgG (H+L) alkaline phosphatase-conjugated secondary antibody (Promega). Positive fractions were developed with Western Blue stabilized substrate for alkaline phosphatase (Promega). Positive fractions were run on sodium dodecyl sulfate (SDS-PAGE) and silver stained using SilverQuest (Invitrogen). Imidazole was removed from positive fractions by dialysis overnight against PBS, pH 7.4.

### Western blotting using human, dog, and fox sera

Salivary glands (40 pairs approximately equivalent to 40 µg total protein) or soluble recombinant sand fly salivary proteins (20 µg) were run on a 4–20% Tris-glycine gel or on a 4–12% NuPAGE gel. After transfer to a nitrocellulose membrane using the iBlot device (Invitrogen), the membrane was blocked with 3% (w/v) nonfat dry milk in Tris-buffered saline (TBS)-0.05% Tween, pH 8.0, overnight at 4°C. After washing with TBS-T, pH 8.0, the membrane was placed on a mini-protean II multiscreen apparatus (Bio-Rad, Hercules, CA), and different lanes were incubated with various sera (1∶80 dilution, human and dog sera; 1∶50 dilution, fox sera) for 3 h at room temperature. After washing with TBS-T, pH 8.0, three times for 5 min, the membrane was incubated with either anti-dog IgG (H+L) alkaline phosphate-conjugated antibody (1∶10,000) (Jackson Immuno Research) for 1 h at room temperature for dog and fox sera or with anti-human IgG alkaline phosphate-conjugated antibody (1∶8,000) (Sigma) for human sera. Membranes were developed by addition of Western Blue stabilized substrate for alkaline phosphatase (Promega), and the reaction was stopped by washing the membrane with deionized water.

## Results

### Recognition of salivary proteins from the sand fly *Lu. longipalpis* by sera from different hosts

**Figure 1:**
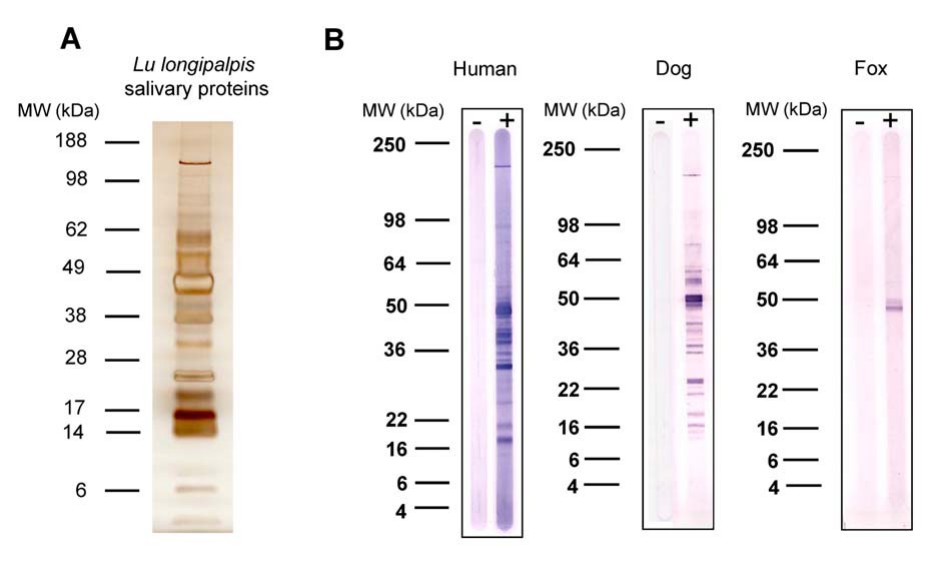
*Lutzomyia longipalpis* salivary proteins recognized by human, dog and fox sera from the visceral leishmaniasis endemic areas of São Luis and Teresina. *A*, Silver staining of a Bis-Tris NuPAGE gel of salivary proteins from *Lu. longipalpis*. *B*, Western Blot of salivary proteins of *Lu. longipalpis* separated on a Tris-Glycine gel using a representative reactive sera from either human (São Luis), dog or fox (Teresina).

*Lu. longipalpis* salivary glands contain a large number of secreted proteins (figure 1A). Fourteen human sera from individuals living in São Luis, an area where *Lu. longipalpis* predominates, recognized a considerable number of these proteins, mainly between 15 and 65 kDa (figure 1B). Eight dog sera from Teresina recognized a large number of salivary proteins, many of the same size as human sera as well as several proteins of different sizes (figure 1B). As for foxes, 11 sera collected in the same endemic area as dogs recognized only a few salivary proteins and only one strongly of approximately 50 kDa (figure 1B).

### Host-specific recognition of *Lu. longipalpis* salivary proteins

**Figure 2:**
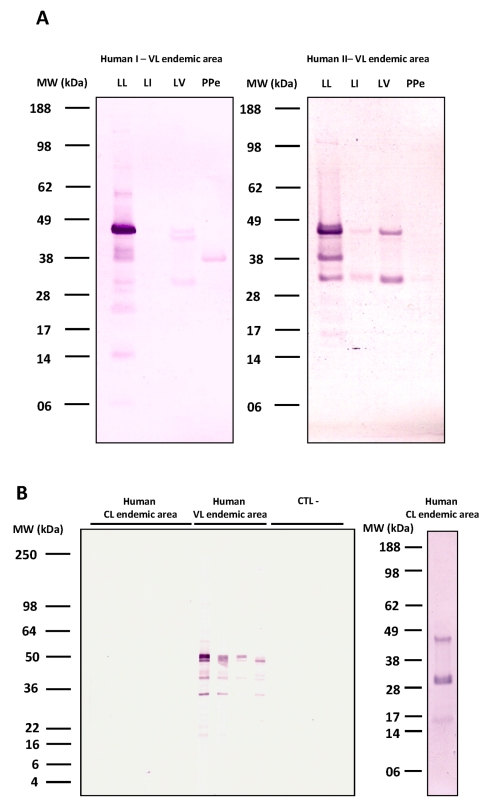
Detection of salivary proteins from different species of sand flies by Western Blot. *A*, Two representative human sera from São Luis, a VL- endemic area where *Lutzomyia longipalpis* predominates, were screened against salivary proteins from *Lu. longipalpis* (LL), *Lu. intermedia* (LI), *Lu. verrucarum* (LV), and *Phlebotomus perniciosus* (PPe). *B*, Western blot analysis of salivary proteins from *Lu. longipalpis* using human sera from Canoa, a cutaneous leishmaniasis endemic area where *Lu. intermedia* predominates (CL endemic area; left bottom panel), or from São Luis where *Lu. longipalpis* predominates (VL endemic area). Sera from individuals from a non-endemic area were used as negative controls (CTL–). The profile of salivary proteins from *Lu. intermedia* recognized by a representative human serum (out of six tested ) from the Canoa endemic area (CL endemic area; right bottom panel).

To determine the specificity of human and dog sera for *Lu. longipalpis* salivary proteins, we tested the most reactive human (two) and dog (one) sera against salivary proteins from other sand fly species including *Lu. intermedia* which transmits CL in South America [24], *Lu. verrucarum* which transmits CL in Central and South America [25], and *Phlebotomus perniciosus* which transmits VL in Mediterranean countries [26]. Human sera recognized multiple bands of *Lu. longipalpis* saliva (figure 2A). One of the 2 human sera also recognized two salivary proteins from *Lu. intermedia* (figure 2A). We cannot exclude the possibility that this individual was weakly exposed to *Lu. intermedia* bites, as this species is also present, albeit rare, in São Luis [20] or to other non-abundant species in the area. All tested sera recognized proteins between 28 and 50 kDa from *Lu. verrucarum* saliva and a protein of approximately 40 kDa from *P. perniciosus* saliva (figure 2A); both species do not overlap with *Lu. longipalpis* in their geographical distribution.

Given that *Lu. longipalpis* and *Lu. intermedia* are sympatric in several areas of Brazil [27] we decided to further investigate the possibility of antibody cross-reactivity between these two species. To address this, we tested six human sera from Canoa (an area in Brazil endemic for CL where *Lu. intermedia* predominates) against *Lu. longipalpis* saliva. These sera did not recognize any of the salivary proteins of *Lu. longipalpis* but recognized those of *Lu. intermedia* (figure 2B). Together, these results suggest an overall low level of cross-reactivity between *Lu. longipalpis* and *Lu. intermedia* salivary proteins.

**Figure 3:**
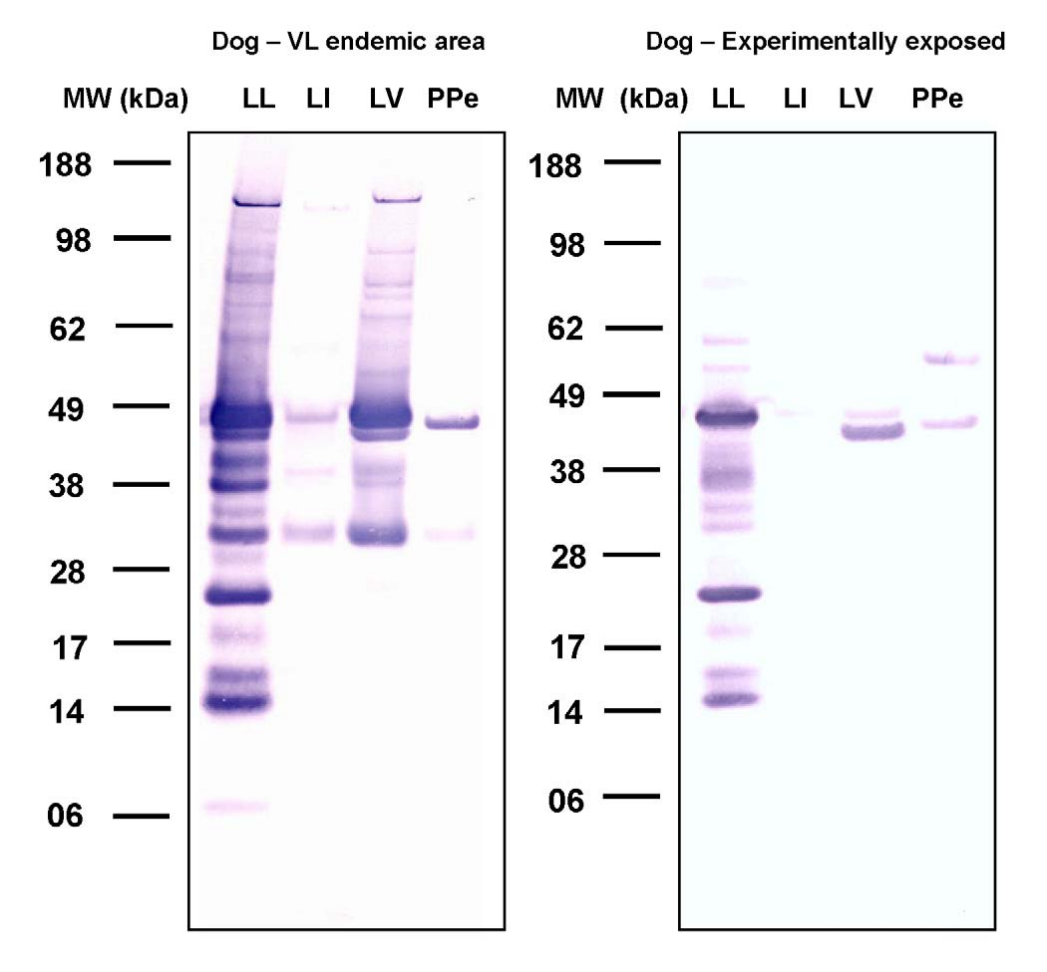
Total IgG response in dogs from endemic areas and dogs experimentally exposed to *Lutzomyia longipalpis* bites. Dog sera from Teresina, an area endemic for visceral leishmaniasis where *Lu. longipalpis* predominates, (Dog-VL endemic area) and sera from dogs experimentally exposed to *Lu. longipalpis* bites (Dog – Experimentally exposed) were screened against salivary proteins from *Lu. longipalpis* (LL), *Lu. intermedia* (LI), *Lu. verrucarum* (LV), and *Phlebotomus perniciosus* (PPe) by Western Blot.

Because in an endemic area there is no control of the diversity and intensity of exposure of hosts to sand fly bites—both of which can influence antibody response [14]—we compared dogs from Teresina, where *Lu. longipalpis* is prevalent, with dogs experimentally exposed to *Lu. longipalpis* bites. Overall, the reactivity of sera from experimentally exposed dogs was considerably lower than that of dogs from Teresina. Both dogs from Teresina and experimentally exposed dogs recognized proteins between 15 to 65 kDa (figure 3). Both groups recognized multiple proteins in *Lu. longipalpis* saliva but also a few in the saliva of *Lu. verrucarum* and *P. perniciosus*. This, together with results from human sera, suggests that antibodies against these proteins may be cross reactive for these two species. Additionally, while proteins from 28 to 50 kDa from *Lu. intermedia* were recognized by sera of dogs from Teresina, only one protein was poorly recognized by sera from experimentally exposed dogs. Sera from foxes were also tested but showed no cross-reactivity with the other species (data not shown).

### Production of soluble recombinant salivary protein in a heterologous mammalian expression system

**Figure 4:**
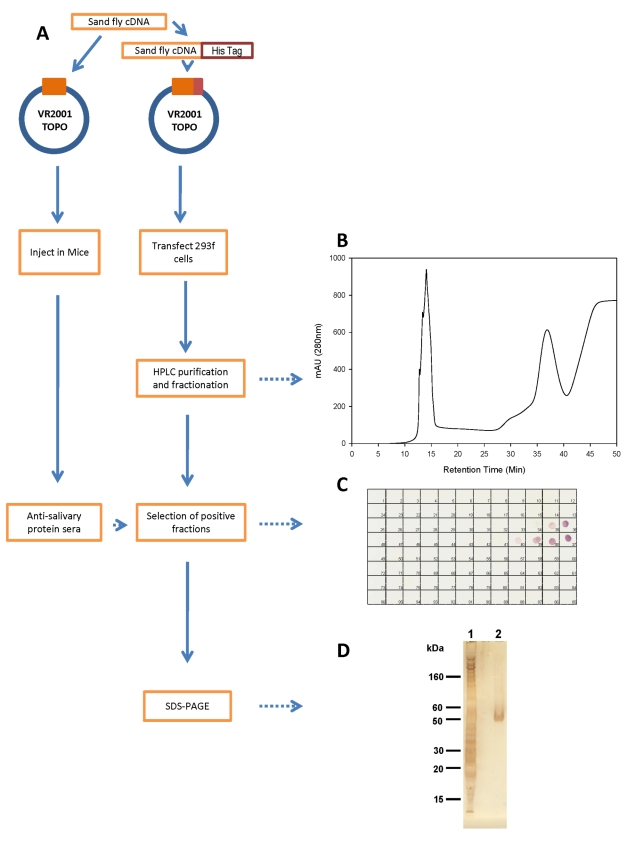
Production of recombinant sand fly salivary proteins. *A*, Flowchart describing the expression, purification and detection of recombinant sand fly salivary proteins from *Lutzomyia longipalpis*. *B*, Chromatogram of the purification of the LJM17 recombinant protein by HPLC-metal affinity chromatography. *C*, Detection of positive fractions for the LJM17 recombinant protein using sera from mice immunized with LJM17 plasmid. *D*, Silver-stained SDS-PAGE of LJM17 recombinant protein before (1) and after (2) HPLC purification.

Nine abundant transcripts corresponding to the predicted molecular weight of the most antigenic salivary proteins recognized by human, dog, and fox sera within the range of 15 to 65 kDa (figure 1) were selected for expression (Table 1). Figure 4 shows a flow diagram of the approach used to express and purify the nine chosen recombinant salivary proteins. Notably, the same DNA plasmid is used for recombinant protein expression and antibody production. Nine different salivary proteins were expressed and a high level of purification was achieved by HPLC. Purification of recombinant salivary protein LJM17 resulted in a well separated peak eluting at 35–40 min (figure 4B). Aliquots of eluted fractions were recognized by sera of mice immunized with LJM17 DNA plasmid (figure 4C). SDS-PAGE of positive fractions shows a single band of approximately 50 kDa (figure 4D), the expected size predicted by the LJM17 transcript. Similar results were obtained with the other eight expressed proteins: LJM111, LJM11, LJL143, LJL13, LJL23, LJM04, LJL138, and LJL11 (data not shown).

**Table 1:** Transcripts selected from a *Lutzomyia longipalpis* salivary gland cDNA library based on predicted molecular weight of secreted proteins.

Transcript	Predicted molecular weight	Annotation	NCBI accession number
**LJM17**	45.2	Yellow related protein	AF132518
**LJM11**	43.2	Yellow related protein	AY445935
**LJM111**	43.0	Yellow related protein	DQ192488
**LJL23**	35.1	Apyrase	AF131933
**LJL13**	26.5	D7 related protein	AF420274
**LJM04**	13.8	SL1 protein	AAD32197.1
**LJL138**	43.7	Endonuclease	AY455916
**LJL11**	60.5	5^/^nucleotidase	AF132510
**LJL143**	32.4	Unknown	AY445936

### Characterization of *Lu. longipalpis* immunogenic proteins

**Figure 5:**
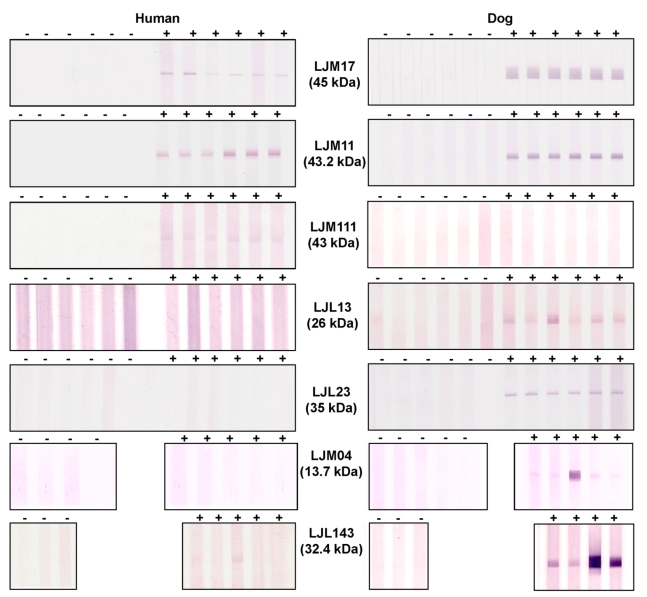
Detection of *Lutzomyia longipalpis* recombinant salivary proteins by human and dog sera from visceral leishmaniasis endemic areas. Human and dog sera from São Luis and Teresina respectively, were used to screen recombinant salivary proteins LJM17, LJM11, LJM111, LJL13, LJL23, LJM04, and LJL143 by Western Blot (+). Human and dog sera from a non-endemic area were used as negative controls (–).

To determine whether the nine expressed salivary recombinant proteins were recognized by sera from humans, dogs, and foxes, we chose those that recognized a considerable number of proteins (from total sand fly saliva) with some degree of variability for further analysis by western blot. Of the nine recombinant proteins tested, LJM17, a yellow-related protein of 45 kDa, was the only protein recognized by sera from the 3 different hosts (figure 5; data for foxes not shown). LJM11, a 43-kDa protein also of the yellow family of proteins, was recognized by human and dog sera, while a third yellow-related protein, LJM111 (43 kDa) was only recognized by human sera (figure 5). LJL23, LJL13, and LJM04 proteins were recognized only by dog sera; LJL143 was recognized by dog sera and weakly recognized by human sera (figure 5). LJL11 and LJL138 were not recognized by any of the sera tested (data not shown).

**Figure 6:**
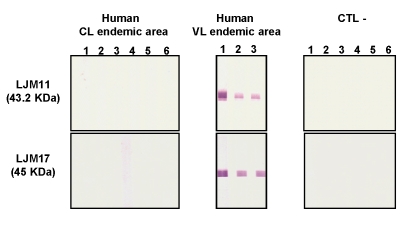
Detection of the recombinant salivary proteins LJM17 and LJM11 by human sera from endemic areas. Western Blot with LJM17 or LJM11 recombinant proteins were screened with sera from humans in Canoa where *Lutzomyia intermedia* predominates ( Human – CL endemic area) or with sera from São Luis area where *Lu. longipalpis* predominates (Human – VL endemic area). Sera from individuals from a non-endemic area were used as negative controls (CTL–).

To confirm the specificity of LJM17 and LJM11 as potential markers of *Lu. longipalpis* exposure, we tested human sera from São Luis and Canoa where *Lu. longipalpis* and *Lu. intermedia* predominate, respectively. Both LJM17 and LJM11 were recognized specifically by human sera from São Luis but not from Canoa (figure 6).

## Discussion

Among parasitic diseases, leishmaniasis has one of the most complex epidemiologies. There are numerous *Leishmania* species and some cause a wide range of clinical manifestations and involve a large number of proven and potential reservoir hosts. In most cases, each form of leishmaniasis is transmitted by a sand fly species that acts as a principal vector; a few Leishmania species have multiple vector species [28]. In addition, endemic areas of leishmaniasis support several sand fly species other than the vector species responsible for Leishmania transmission. Finding tools to measure exposure of humans and reservoir hosts to specific vectors would provide valuable information regarding their contribution to parasite transmission and would be useful for assessing the risk of contracting disease.

Several studies have demonstrated that anti-saliva antibodies can be used to assess exposure of humans and other *Leishmania* hosts to sand fly bites and suggested that sand fly salivary proteins represent attractive targets for development of specific markers of vector exposure [4],[14],[16]. To date, none of the salivary proteins of sand flies have been characterized for their immunogenicity and specificity in mammalian hosts, an important prerequisite for their reliability as markers of exposure.

In the present work, we developed a robust method for producing and purifying recombinant salivary proteins. This approach proved highly successful in obtaining pure recombinant proteins that retain recognition epitopes. The purity of the produced recombinant salivary proteins is demonstrated by the presence of a single protein band following SDS-PAGE and silver staining; this level of purity was obtained for all recombinant salivary proteins tested. Soluble and pure recombinant proteins are likely to be properly folded and to better resemble native proteins. This should improve the sensitivity of the detection, enhancing recognition of such proteins by test sera and increasing the specificity of a test by decreasing the chances of false negatives often caused by impurities in the preparation. It is worth noting that the tested salivary recombinant proteins demonstrated specific responses, as two (LJL11 and LJL138) of the nine proteins were not recognized by any of the tested sera.

The seven immunogenic recombinant salivary proteins were differentially recognized by human, dog, and fox sera, the three host species investigated. Some proteins displayed host-specific recognition, reinforcing the importance of testing potential salivary markers for exposure against a variety of hosts to determine their range of applicability. Recombinant proteins LJM17 and LJM11 were strongly recognized by human sera from São Luis, a VL endemic area where *Lu. longipalpis* predominates, representing 66.4% of captured sand flies [29]. Notably, human sera obtained from Canoa, a CL area where *Lu. intermedia* represents 94% of the sand fly population [22], did not recognize LJM17 or LJM11. *Lu. longipalpis* and *Lu. intermedia* are sympatric species in many endemic areas of Brazil, often representing the two most abundant sand fly species [27]. As such, LJM17 and LJM11 can be considered as potential specific markers of exposure to *Lu. longipalpis* in areas where other man-biting sand fly species are present in negligible numbers [20],[24],[27]. LJM17 and LJM11 should be tested for specificity against other man-biting sand fly species that are relatively abundant in endemic areas—such as *Lutzomyia whitmani* [29]—to expand their utility as specific markers of exposure to *Lu. longipalpis*. Indeed, the faint bands recognized in *Lu. intermedia* saliva against one reactive human serum from São Luis (figure 2) may have been due to cross reactivity between salivary proteins of this species and those of *Lu. whitmani*, reported to constitute as much as 24% of the sand fly population in this area [29]. The absolute specificity of LJM17 and LJM11 for *Lu. longipalpis* exposure therefore requires further confirmation through studies that target more sand fly species. Serum samples from São Luis also recognized salivary proteins from *P. perniciosus*, a vector of VL in the Old World [30]. This is interesting, as sera from inhabitants of Sanliurfa, Turkey, where *P. papatasi* and *P. sergenti*—two established Old World CL vectors—are abundant, did not react with salivary proteins of *Lu. longipalpis* [16].

Both LJM17 and LJM11 were recognized by dog sera from Teresina, endemic for canine VL [19]. A recent survey of the sand fly population from this area showed that *Lu. longipalpis* represented 99.7% of the collection [20]. Different from work done previously, here we detected six different salivary proteins as potential specific markers for exposure to *Lu. longipalpis* by using recombinant proteins for dogs (figure 5). The dog-biting status of other *Lutzomyia* species needs to be established before the specificity of these proteins as markers of exposure to *Lu. longipalpis* can be validated. However, this does not detract from their usefulness as potential markers for sand fly exposure for the evaluation of intervention studies in dogs.

Although we did not test potential cross-reactivity of LJM17 and LJM11 with salivary proteins from other common vectors such as mosquitoes, kissing bugs, and black flies, extensive comparative transcriptomic analysis confirm that these two proteins are unique and distinct from those in the saliva of other arthropod vectors [31]–[35].

In conclusion, we have identified two salivary proteins from *Lu. longipalpis*, LJM17 and LJM11, that were specifically recognized by sera from humans living in an endemic area of VL. Once tested on a wider scale, these proteins could become an important tool for accurate surveillance of this important vector of VL in Latin America.
